# Development of Films for Wound Healing Based on Gelatin and Oil/Water Emulsions as Carriers of Bioactive Compounds

**DOI:** 10.3390/pharmaceutics17030357

**Published:** 2025-03-11

**Authors:** Ayelen M. Sosa, Celeste Cottet, Belén E. Berin, Luis M. Martínez, Mercedes A. Peltzer, María J. Prieto, Carolina S. Martinez

**Affiliations:** 1 Laboratorio de Bio-Nanotecnología (LBN), Departamento de Ciencia y Tecnología, Universidad Nacional de Quilmes, Bernal 1876, Argentina; beleneliana30@gmail.com (B.E.B.); lmartinez@unq.edu.ar (L.M.M.); jprieto@unq.edu.ar (M.J.P.); carolina.martinez@unq.edu.ar (C.S.M.); 2 Grupo de Biología Estructural y Biotecnología, Instituto Multidisciplinario de Biología Celular, Consejo Nacional de Investigaciones Científicas y Técnicas, CICPBA, Universidad Nacional de La Plata, La Plata 1900, Argentina; 3 Laboratorio de Obtención, Modificación, Caracterización y Evaluación de Materiales (LOMCEM), Departamento de Ciencia y Tecnología, Universidad Nacional de Quilmes, Bernal 1876, Argentina; celestecottet@gmail.com (C.C.); mercedespeltzer@gmail.com (M.A.P.); 4 Consejo Nacional de Investigaciones Científicas y Técnicas (CONICET), Godoy Cruz 2290, Argentina

**Keywords:** gelatin films, silver nanoparticles, o/w emulsions, characterization

## Abstract

**Background:** Natural biopolymeric matrices for developing dressings have been extensively studied, as they may exhibit beneficial properties for wound healing. Gelatin possesses promising structural and physicochemical properties for incorporating active compounds (ACs). O/W emulsions are an alternative delivery system for AC with different properties and solubilities, promoting wound healing. **Objective:** This study aimed to develop gelatin films by adding silver nanoparticles and healing agents encapsulated in an O/W emulsion to treat skin wounds. **Methods:** A film-forming dispersion was prepared using gelatin and glycerol as a plasticizer, and films were obtained using the casting technique. Emulsions with ACs (EAs) and without ACs (ECs) were incorporated into the films. The formulations were analyzed by FESEM and characterized for their mechanical, thermal, and swelling properties, as well as their water vapor permeability. **Results:** The results showed that films with a higher amount of emulsion exhibited greater structural rigidity and lower permeability, while films with lower amounts of emulsion demonstrated more elasticity and higher permeability. General and organ-specific toxicity were evaluated in zebrafish larvae. The films showed no lethal or sub-lethal effects on the morphology or activity of the brain, heart, and liver. **Conclusions:** The active films developed could provide stable support and a safe delivery system for active compounds to treat skin lesions, minimizing the risk of infection and the need to heal a wound.

## 1. Introduction

Using natural biopolymers for biomedical and pharmaceutical applications has various implications, including developing scaffolds for drug delivery, wound healing, and tissue engineering, owing to their gelation capacity and high availability in nature [1]. Notably, some biopolymers have good film-forming capacities, such as chitosan, starch, alginate, carrageenan, gelatin, and pectin [2,3] Gelatin is a high-molecular-weight polypeptide, as well as an important hydrocolloid used in various fields due to its functional characteristics, which include water retention ability, gel formation, film formation, foam-forming ability, and emulsification tendency [4,5]. Its structure comprises chains of amino acids that form a three-dimensional network held together by hydrogen bonds and hydrophobic interactions, making it a multifunctional biomaterial [6]. Furthermore, collagen is a protein that constitutes the extracellular matrix (ECM) and plays a vital role as connective tissue in biological structures, serving as the source of elastin and contributing to the tissue’s structural and physiological integrity [7]. This is why it has become relevant in developing human skin wound dressings and biomimetic materials.

Silver nanoparticles (AgNPs) play a fundamental role in nanotechnology for their versatile applications in the medical, cosmetic, and pharmaceutical industries. Its biocidal properties at the nanoscale are attributed to the high fraction of surface atoms per unit of volume, which is responsible for its activity [8,9]. As with any nanoparticles, the use of AgNPs needs to be thoroughly analyzed and regulated to protect the environment [10]. Previous studies showed the effectiveness of AgNPs as antimicrobial agents in in vitro studies against *Staphylococcus aureus* and *Pseudomonas aeruginosa.* In addition, their potential toxicity was evaluated in zebrafish embryos and larvae, and a spectrum of AgNP concentrations with good antimicrobial activity and no biotoxicity were found [11]. In addition to antimicrobial agents, it is crucial to incorporate active ingredients that promote wound healing, either through direct effects or by creating a suitable environment for the body to respond to the presence of an injury. Examples of such components include vitamin A, lidocaine, and sulfadiazine. Vitamin A, or retinol, is an antioxidant component that promotes cell regeneration through direct interaction with cells or hormones that stimulate wound re-epithelialization [12]. Lidocaine is an anesthetic component that plays a role in signaling mechanisms that promote skin regeneration, such as directing neutrophils and monocytes to the wound site [13]. Finally, sulfadiazine acts as a second antimicrobial component, exerting a synergistic effect with AgNPs to prevent potential complications from microorganism infections [14].

The design of nano- and microstructured carrier systems for encapsulating active compounds is an attractive strategy for developing targeted therapies. These types of carriers, such as liposomes, nanoparticles, dendrimers, and emulsions, must be carefully designed because their structural characteristics will determine not only their pharmacokinetic and biodistribution properties but also those of the active compounds encapsulated [15,16]. Medical and cosmetic industries are very interested in O/W emulsion development for the encapsulation of diverse active compounds [17], and most of the applications are nutraceutical formulations rich in ω6 and ω9 [18,19]. An interesting development for epidermal treatments involved the use of films with O/W emulsions, as they combine the structural advantages of biopolymers for a steady superficial matrix and the delivery advantages of hydrocolloid systems [20,21]. Indeed, the lipid component present in the emulsion can act as an excellent barrier, reducing the exchange of water vapor, oxygen, and carbon dioxide between the skin lesion and the environment [22].

This work aimed to develop and characterize gelatin films by adding silver nanoparticles and healing agents to treat skin wounds. This development involves the incorporation of active agents with different properties and solubilities into a single film, potentially addressing the needs of an organism during the healing process of an injury. Additionally, the toxicological effects will be studied using the zebrafish model, providing insights into the potential impact of this development on living organisms.

## 2. Materials and Methods

### 2.1. Materials

The colloidal suspension of AgNPs was provided by Nanotek S.A. (nanoArgen, Acassuso, Argentina). Sulfadiazine (Sigma-Aldrich, St. Louis, MO, USA, EE.UU, CAS-No: 68-35-9), lidocaine (Sigma-Aldrich, St. Louis, MO, USA, CAS-No: 137-58-6), propylene glycol (BioPak, Lanús, Argentina, CAS-No: 57-55-6), gelatin (Sigma-Aldrich, St. Louis, MO, USA, CAS-No: 9000-70-8), tween 80 (BioPak, Lanús, Argentina, CAS-No: 9005-65-6), and glycerol (Anedra Research A.G., Buenos Aires, Argentina, CAS-No: 56-81-5) were acquired from their corresponding suppliers. Olive oil, canola oil, vitamin E, vitamin A, and soy lecithin were obtained from the local pharmacy and were suitable for human intake.

### 2.2. Preparation of Gelatin Films with Emulsions (O/W)

The emulsions were prepared by mixing an oil phase (cod liver oil, vitamin E, and canola oil) with a mix of surfactant agents: Tween 80, soy lecithin, and propylene glycol (PPG); active ingredients (silver sulfadiazine, lidocaine, and vitamin A; continuous phase: silver nanoparticles (AgNPs) and distilled water), using the spontaneous emulsification method following the protocol described by [23]. The proportions of the components of the oil phase and the mix correspond to those previously published by [18,24]. An emulsion containing the active ingredients in its composition (AE) was prepared, along with an emulsion without active ingredients (CE). The AE was formulated by mixing the active compounds in a 1:1:0.25 mass ratio within the oil phase. In addition, a mixture of surfactant and AgNPs (sized between 4 and 7 nm) was prepared by dispersing AgNPs in water at a ratio of 0.05:1 (AgNPs/water) and adding them dropwise to the oil phase at room temperature under vigorous stirring. The CE was prepared similarly to the AE but using a 1:1 ratio of water to PPG as the active ingredient. The emulsions were stored at room temperature and protected from light to prevent decomposition until the moment of their use.

The film solution was prepared using gelatin as the biopolymer at 3% *w*/*v* in distilled water and glycerol as a plasticizer at 1.5% *w*/*w* relative to the amount of gelatin. The gelatin was dissolved in distilled water once it reached 90 °C, and from that moment, it was homogenized for 20 min by continuous stirring. The active emulsion and the control emulsion were added to the film-forming dispersion in two different ratios: 5:1 (AE_5_/CE_5_) and 10:1 (AE_10_/CE_10_) (parts of film solution to parts of emulsion, respectively). The resulting dispersion was poured into sterile Petri dishes and left to dry using the casting method at room temperature until it completely dried. A control film was also prepared, composed solely of the polymer (gelatin) and the plasticizer, which we will refer to as “gelatin film (GF)”. For subsequent characterization, the films were conditioned in a desiccator with 53% relative humidity (r.h.) using a saturated Mg(NO_3_)_2_ solution. The nomenclature used throughout the work to identify the different films is described in Table 1.

### 2.3. Physicochemical Characterization

#### 2.3.1. Field Emission Scanning Electron Microscopy (FESEM)

The morphology of the films was examined by FESEM (Zeiss Crossbeam 340). All samples were mounted on stubs horizontally on their surface and coated with gold before FESEM imaging at an accelerating voltage of 3 kV.

#### 2.3.2. Mechanical Properties

Measurements were performed using a Universal Test Instrument Megatest TC-500 series II (Megatest, San Martin, Argentina) equipped with a 30 kgf cell load, following the ASTM D882-12 standard. The experiment was performed at 20 mm min^−1^, at room temperature. Before testing, specimens measuring 50 mm × 10 mm were cut from each formulation, and their thickness was obtained by measuring them at 5 different points with a digital micrometer (INSIZE Co., Ltd., Xiangyang Road, Suzhou, China). The mechanical parameters were calculated from the resulting stress–strain curves: tensile strength (TS, MPa), Young’s modulus (YM, MPa), and elongation at break (ε, %). Three replicates were performed for this test.

#### 2.3.3. Swelling Capacity

The swelling behavior of the films was evaluated in distilled water at room temperature. Film disks (about 7 mg) were cut for each formulation, weighed initially, and submersed in a small container with 10.5 mL of distilled water. At different intervals, samples were removed and carefully dried with filter paper to remove the residual water, weighed, and submerged again. This procedure was performed until constant weight or loss of integrity of the films. The swelling degree was expressed as water uptake, h_(t)_, at time t in units of g of water per 100 g dried film (d.f.) and was calculated following Equation (1):

(1) $$h_{\left(t\right)}=\frac{p_{f}-p_{0}}{p_{0}}\times 100$$

where p_f_ is the weight of a swollen disk at time t and p_0_ is the initial weight of the disk that was measured at the beginning of the experiment. Three replicates were performed for this test.

#### 2.3.4. Water Vapor Permeability (P_w_)

The films’ water vapor permeability (*P_w_*) was measured using the cup method described in ASTM E-96 with some modifications by quantifying the water vapor flux through the film by changes in the system’s weight, owing to moisture transfer. Firstly, films were sealed on the cups with a 53 mm diameter aperture, containing a saturated solution of BaCl_2_ providing 90% r.h. Test cups were placed inside a desiccator containing a saturated solution of NaOH, providing 10% r.h. at a constant temperature of 22 °C. To maintain uniform conditions, a fan was placed inside the desiccator over the films. At the beginning of the experiment, cup weights were recorded, and then weight loss was measured over time using an analytical precision balance (Precisa 125 A SCS, ±10^−4^ g). Weight loss m versus time t was plotted, and when the steady state (straight line) was reached, another 48 h was registered. *P_w_* (g s^−1^ m^−1^ Pa^−1^) was calculated following Equation (2):

(2) $$P_{w}=\frac{1}{A}\left(\frac{\Delta m}{\Delta t}\right)\frac{L}{\Delta p_{w}}$$

where *A* is the effective area of the exposed film, *L* is the film thickness, and Δ*p_w_* = (*p_w_*_2_ − *p_w_*_1_) (in Pa units) is the difference in water vapor partial pressure across the film; *p_w_*_1_ and *p_w_*_2_ are the partial pressures at the film surface outside (263.9 Pa) and inside the cup (2375.4 Pa), respectively. Experiments were performed in duplicate.

#### 2.3.5. Thermal Gravimetric Analysis (TGA)

The thermal stability of the materials was assessed using a thermogravimetric analyzer (TA Instrument Q500, New Castle, DE, USA). Approximately 7 mg of each sample was weighed in a platinum pan and heated from 30 °C to 700 °C under an inert nitrogen atmosphere (flow rates: 40 mL min^−1^ in balance and 60 mL min^−1^ in the sample) at 10 °C min^−1^ to study the thermal degradation. To analyze the behavior of the films at low temperatures, samples were heated from 30 °C to 120 °C at 1 °C min^−1^. The initial degradation temperature (T_i_) was determined at 15% of mass loss, and the temperature at the maximum degradation rate (T_max_) was determined from the peak of the derivative curves of mass loss with respect to temperature. Experiments were carried out in duplicate.

#### 2.3.6. Differential Scanning Calorimetry (DSC)

The glass transition temperature (Tg) and endothermic enthalpy (ΔH) were measured by a Differential Scanning Calorimeter (TA Instruments Q200, New Castle, DE, USA). Approximately 5 mg of each film was contained in hermetic aluminum pans and sealed with hermetic lids. The heating was carried out from 30 °C to 120 °C at 10 °C min^−1^. Tg and endothermal transitions were determined by TA Universal Analysis software (v4.5, TA Instruments, New Castle, DE, USA), and analyses were performed in duplicate.

#### 2.3.7. Fourier-Transform Infrared Spectroscopy with Attenuated Total Reflectance (FTIR-ATR)

The infrared spectra of the gelatin-based films were measured using a Fourier-Transform Infrared Spectrophotometer (FT-IR) (Affinity-1, Shimadzu Co., Kyoto, Japan) fitted with the module GladiATR (Pike technologies, Madison, WI, USA). The spectra were obtained in duplicate, from 400 at 4000 cm^−1^, with an average of 45 scans at a 4 cm^−1^ resolution. Before each test, the humidity and the presence of carbon dioxide in the air were compensated for with a blank spectrum. This test was performed in duplicate.

### 2.4. Cytotoxicity and Wound-Healing Assay

For in vitro assays, immortalized human skin keratinocyte cultures (HaCaT cell line) were used, maintained in RPMI medium supplemented with 10% fetal bovine serum (FBS) and 1% glutamine. To allow cell growth, they were kept in an incubator with 5% CO_2_ at 38 °C. Cytotoxicity assays were performed to determine cell viability using crystal violet (CV), metabolic activity using MTT, and membrane integrity using neutral red uptake (NR). The cytotoxicity study was conducted following the protocol of [11]. For the wound-healing assay, HaCaT cells were grown to confluence in sterile 6-well plates for the cell migration study. A wound was created using a sterile p200 pipette tip, followed by washing with 1× phosphate-buffered saline (PBS). Finally, the cells were incubated with the supernatant from 1.5 cm film disks that had been in contact with the RPMI-supplemented medium for 24 h. After incubation, the cells were fixed with 30% *v*/*v* formaldehyde for 10 min, followed by incubation with 10% *w*/*v* methylene blue. Cell migration was then observed with cytation 5 (Cell Imaging Mµlti-Mode Reader, BioteK, Córdoba, Argentina), and the cell migration rate was measured using ImageJ software V5.1. Each cytotoxicity assay was performed in triplicate, as was the cell migration assay. The migration rate was calculated following Equation (3):

(3) $$Migration\;rate=\frac{\;(wound\;size_{0hpi}-wound\;size_{24hpi})}{24\;h}$$

### 2.5. Toxicology Study in Zebrafish Model

**Figure 1:**
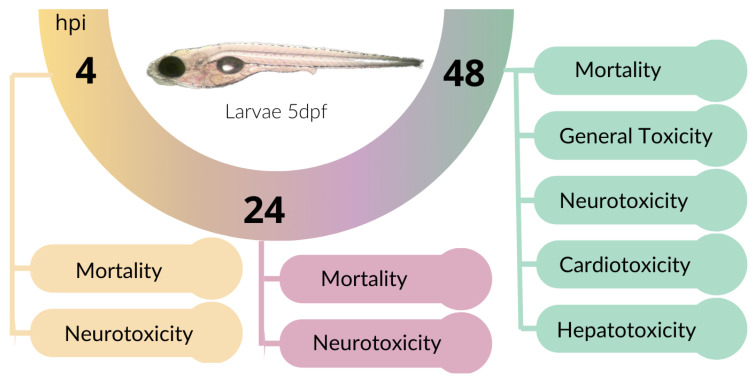
Experimental design. Zebrafish larvae of 5 days post-fecundation (dpf) were incubated with different films. Mortality, locomotor activity (neurotoxicity), cardiac rate (cardiotoxicity), necrotic liver (hepatotoxicity), and morphological changes were studied at 4, 24, and 48 h post-incubation (hpi).

The toxicological study of the developed films was carried out using the zebrafish model. The protocols used were approved by the Institutional Animal Care Committee of the National University of Quilmes, resolution CE-UNQ 2/2014, and Institutional Committee for the Care and Use of Laboratory Animals, with the following resolution CICUAL-UNQ 013-15. General toxicity and organ-specific studies were conducted by incubating 5-day-post-fertilization (dpf) larvae with the supernatant resulting from the incubation of 1.5 cm diameter film disks, previously UV-sterilized for 2 min, in 2 mL of E3 medium. These larvae were incubated for 48 h at 28 °C, during which mortality, morphological changes, cardiotoxicity, neurotoxicity, and hepatotoxicity were evaluated according to [11,25]. The experimental design is summarized in Figure 1.

For the mortality, morphological changes, and neurotoxicity studies, this research included 16 technical replicates and 3 biological replicates for each tested condition (*n* = 48 larvae in total). For the cardiotoxicity and hepatotoxicity studies, 3 replicates were performed, using *n* = 5 for each biological replicate and a total *n* of 15. Technical replicates refer to the number of wells used in each biological replicate, with each well containing 3 larvae.

### 2.6. Statistical Analysis

Statistical analysis was performed with the GraphPad Prism V6 statistical program, using one-way ANOVA or two-way ANOVA tests followed by Dunnett’s or Tukey’s multiple comparison post-test. Data were presented as a mean ± standard deviation; the error was considered statistically significant if *p* < 0.05.

## 3. Results and Discussion

### 3.1. Field Emission Scanning Electron Microscopy (FESEM)

**Figure 2:**
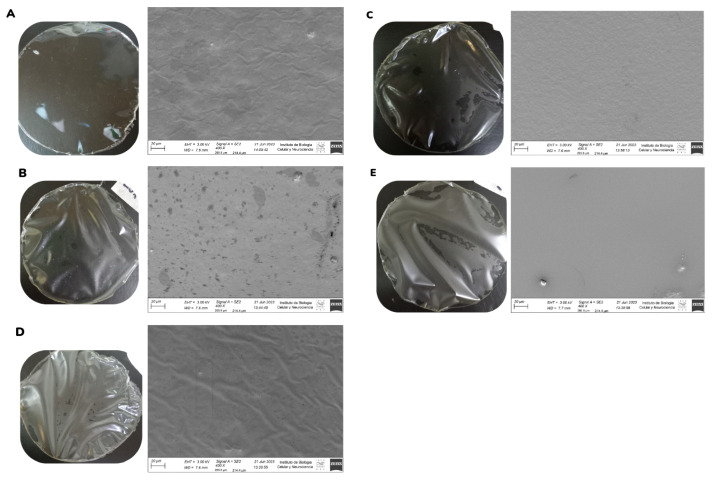
FESEM for gelatin-based films developed at a magnification of 400×. GF (**A**), CE_10_ (**B**), CE_5_ (**C**), AE_10_ (**D**), and AE_5_ (**E**).

Images of the films and the micrographs obtained by FESEM showed the homogeneous and rough surface of control films (Figure 2A). In contrast, films with incorporated emulsions exhibited a heterogeneous surface with no brittle areas and some bubbles (Figure 2B,E). These results are consistent with the observations made by [26]), who indicated that the incorporation of emulsions into gelatin films generates changes in the uniformity of the structures of the resulting films due to the interaction of the emulsion droplets with the matrix. On the other hand, [27] also observed the presence of rough surfaces when incorporating Pickering emulsions in a gelatin film matrix. Gelatin generates foam when shaken to form the film-forming dispersions and might produce bubbles in the matrix. This could be solved by homogenizing the dispersions at a lower stirring speed; however, longer stirring periods could cause the dissolution of the emulsions, as observed in Figure 2B,E. The spots observed in Figure 2B could be related to the rupture of the emulsion droplets, caused by a destabilization process resulting from their incorporation into the film dispersion. Previous studies indicated that the control emulsion (CE) is less stable than the active emulsion (AE) [23]. Differences in the structures of films could be attributed to the emulsion/film-forming dispersion ratio used in each formulation. Figure 2C,D show films with homogeneous surfaces. The film in Figure 2C exhibits wrinkles in its structure, possibly due to the film’s arrangement when mounted on the stub for microscopy and subsequent metallization.

### 3.2. Mechanical Properties

The use of gelatin in composite films is limited by several factors, including low stability, poor mechanical strength, and low elasticity. Many studies have been carried out to solve these weaknesses by adding different compounds into the formulation. Thus, modifications in gelatin films are essential to minimize those limitations [28]. The values corresponding to tensile strength (TS), Young’s modulus (YM), and elongation at break (ε%) of the different films are presented in Table 2. Films prepared using different blend ratios of gelatin have been found to exhibit mechanical parameter values both higher and lower than those observed in this study. Moreover, comparing data with other research is challenging due to variations in experimental factors such as the source of gelatin (bovine, porcine, chicken, or fish), as well as differences in formulation and film-conditioning techniques [29]. All formulations showed changes in the mechanical properties of films, with an increase in TS, YM, and ε% compared to control (GF). For AE emulsions, an increase in TS, YM, and ε% was observed as the proportion of active compounds was increased in the formulation.

The TS parameter showed higher values when the emulsions were incorporated, increasing by 80% to 182% compared to GF. TS is related to the hardness of the material structure, and it was observed that this increased with the addition of the emulsions, indicating that the structures offer excellent resistance to deformation. Additionally, YM exhibited higher values when the emulsions were incorporated into the gelatin matrix. A general analysis of YM across the formulations ranked them as AE_5_, CE_5_, AE_10_, CE_10_, and GF, indicating that the structures provide greater rigidity in the elastic zone. Similarly, the ε% values increased with the presence of the emulsions in the formulations, ranked as CE_10_, CE_5_ ≈ AE_5_ ≈ AE_10_, and GF. This suggests that the structures demonstrate enhanced deformability throughout the tensile-specific deformation curve. The higher ductility of the material was reflected in the total deformation during both the elastic and plastic phases, shown by the incremental values with the addition of the emulsion. The incorporation of the emulsion may have a plasticizing effect, as the emulsion droplets interfere with the formation of intermolecular junctions between peptide chains [30]. However, the interaction between polymer chains was also observed by the increase in material resistance. This behavior could be attributed to the breakage of some emulsion droplets, releasing oil into the matrix and increasing the interaction between hydrophilic chains. Studies have observed similar trends while incorporating liquid oils into hydrophilic biobased films [31,32].

### 3.3. Swelling Capacity

**Figure 3:**
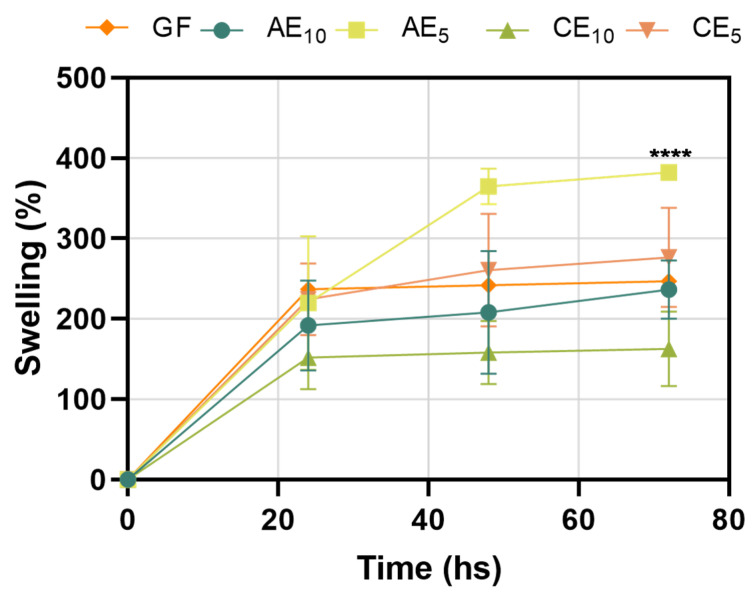
Swelling curves of each film. For statistical analysis, the results are presented as mean ± SEM. The statistical analysis was performed using two-way ANOVA and Tukey’s multiple comparison test (**** *p* < 0.0001).

The water absorption capacity of the different films can be seen in Figure 3; for all conditions studied, a progressive increase in the swelling degree of the films was observed due to the water retained in their structure.

Based on the results, it was possible to identify that the AE_5_ and CE_5_ conditions incorporated more water in their structure than the AE_10_ and CE_10_ conditions and that the GF is located at intermediate points of the tested samples. The ability of the films to incorporate water depends on the physicochemical factors of the medium, the matrix, or its modification due to the incorporation of different components [33,34,35]. In this study, the absorption capacity changed as the structure of the base film was modified. The characteristics of the films mentioned before would help the absorption of exudates found in wounds when suppurating, as a result of an injury and subsequent release of bactericidal and healing components, promoting cell regeneration [7]. The swelling capacity of the films did not exceed 400% of their initial weight in the tested conditions, being able to maintain their structure without breaking or losing integrity; notably, the assay was performed in extreme conditions since a wound would not exude as much liquid. After 72 h of incubation, the films reached equilibrium; they did not incorporate more water into their structure, indicating their maximum swelling capacity. Differences were not significant for most conditions, except for the AE_5_ sample that showed more water absorption capacity.

### 3.4. Water Vapor Permeability

An ideal wound dressing should provide a proper environment for the healing process. Therefore, it must have specific properties, such as flexibility, adherence, and permeability to water vapor [7]. The water vapor permeability (Pw) data are presented in Table 3. Some studies have reported that, for hydrophilic films, water vapor permeability increases significantly with the increasing thickness of the films under study [22]. In this work, Pw increased significantly for gelatin films with emulsion in the formulations compared to the control. When comparing results for films with emulsion, it was observed that higher amounts of emulsion (AE_5_/CE_5_) resulted in slightly higher Pw values. However, their thicknesses were greater than those of the AE_10_/CE_10_ samples, so it was not possible to conclude that the emulsion increased the Pw of the films. When films with equal concentrations of emulsion were compared (same thickness), it was noted that the presence of the active compound slightly increased the permeability value (though not significantly), likely due to the release of some oil from the emulsion without the active compound, which made the matrix more hydrophobic. Other studies have demonstrated that emulsions with active compounds are more stable than those without [11]. Regarding the required Pw for wound dressing, it has been reported that commercial dressings, such as Biobrane (silicon and nylon), have a value of 23 g/m^2^·h·kPa, Omiderm (polyurethane) 83 g/m^2^·h·kPa, and Op-site (breathable dressing and waterproof) 8 g/m^2^·h·kPa. The results of this study, expressed in the same units, ranged between 13 and 17 g/m^2^·h·kPa, indicating that they are adequate for such applications in terms of Pw and represent a sustainable alternative.

### 3.5. Thermal Gravimetric Analysis (TGA)

**Figure 4:**
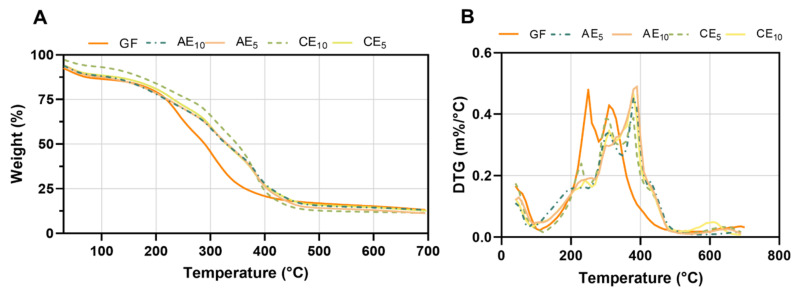
Thermogravimetric curves. (**A**) Weight loss as a function of temperature and (**B**) derived from the weight percentage as a function of temperature.

Thermograms analyzed by TGA scanned between 30 °C and 700 °C showed that the films suffered a multistep degradation (Figure 4), with a weight loss as a function of temperature (Figure 4A) and the derivate of the percentage of weight as a function of temperature (Figure 4B). The main temperatures that occurred during film degradation are described in Table 4. Thermogravimetric analyses showed a displacement of initial temperature (T_i_) in all conditions with respect to GF. This initial temperature was calculated as a 15% weight loss. Then, T_max_ was determined as the temperature peak from the derivate curve (Figure 4B). GFs presented two main peaks centered at 280 °C and 305 °C, but formulated films presented three main peaks that appeared at temperatures near 300 °C, 380 °C, and 440 °C (Figure 4B). Formulated samples containing emulsion exhibited a decrease in the degradation rate compared to those containing GF, and this demonstrated that the emulsion incorporation results in increased thermal stability [27,36,37] in agreement with other authors. Then, a comparison between AE and CE conditions revealed that films under AE displayed lower degradation temperatures than those under CE. This phenomenon may be elucidated by the presence of metallic particles in the formulation, which may serve as internal heat sources within the film, internally increasing the temperature throughout the study and thus facilitating the degradation process.

### 3.6. Differential Scanning Calorimetry (DSC)

**Figure 5:**
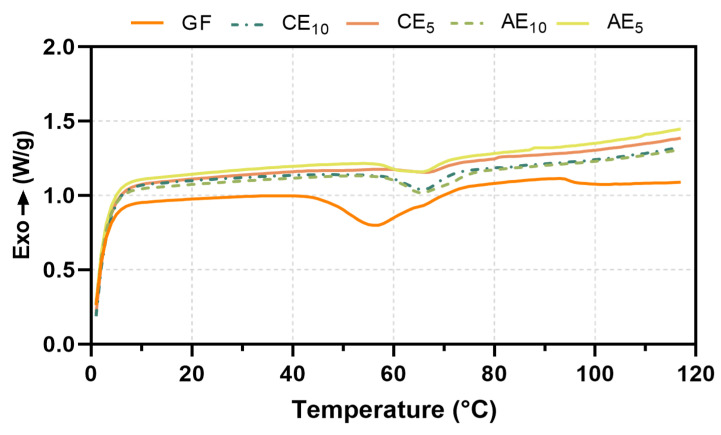
Thermograms of film samples.

DSC thermograms of the different formulations can be observed in Figure 5. From the graph, it was possible to determine the glass transition temperature (Tg) and the endothermic transition (ΔH) corresponding to the denaturation of collagen triple-helix structures and the denaturation temperature (Td) [38,39].

As shown in the DSC thermogram (Figure 5) and Table 5, a shift of the Tg towards higher temperatures of the CE and AE films with respect to the GFs was observed. This behavior could be attributed to the emulsions’ incorporation, which might hinder gelatin chain movements, increasing this temperature. However, the incorporation of the active principles did not play a preponderant role in this behavior since both AE and CE behave similarly under the same conditions. 

The increase in the formulated samples’ enthalpy indicated that the emulsion formulations require more energy to undergo the phase transition. On the other hand, the AE formulations presented higher ΔH values than the CE formulations, implying that the emulsion’s bioactive compounds stabilize the samples.

### 3.7. Fourier-Transform Infrared with Attenuated Total Reflectance (FTIR-ATR)

**Figure 6:**
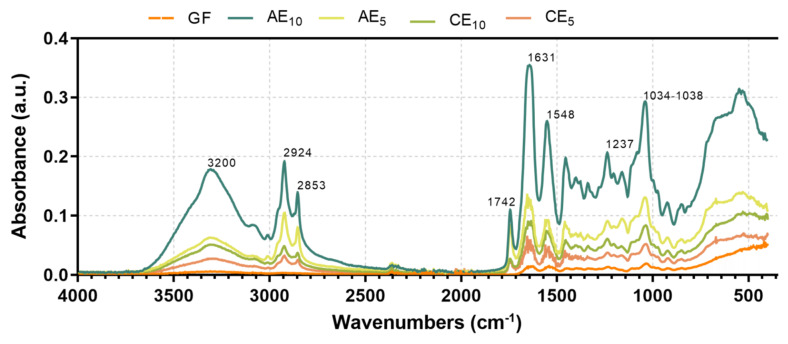
Fourier-Transform Infrared Spectrum with Attenuated Total Reflectance (FTIR-ATR) of gelatin-based films.

The spectrum of the GF and all formulations are shown in Figure 6. All spectra were similar in the interval of 1700–700 cm^−1^, which correspond to the amide I at ~1631 cm^−1^ (C=O stretching/COO-coupled hydrogen bond), amide II at ~1548 cm^−1^ (bending vibration of the N-H group and C-N stretching), and amide III at ~1237 cm^−1^ (vibrations in the C-N and N-H planes of bound amides and CH_2_ to glycine) [40,41]. The band from 1034 to 1038 cm^−1^ in all samples corresponds to the OH groups from glycerol. An increase in the height of the peaks of the amides and the hydroxyl groups with respect to those of the based films was observed, which corresponds to interactions between the structural polymer and the components of the emulsions. The absorption bands between 2853 and 2924 cm^−1^ were attributed to lipids and hydrophobic substances in the emulsion’s active compound and oil phase. Also, the region at 1200–900 cm^−1^ was attributed to stretching modes of carbohydrate molecules and side groups (C-O-C, C-OH, and C-H) and ~1742 cm^−1^, corresponding to the carbonyl group from fatty acids coming from the emulsified oil [42]. At higher wavelengths, the signals found at 3200–3500 cm^−1^ correspond to the vibrational stretches of O-H and N-H groups from the hydrogen bonds within the matrix chains (gelatin) [43,44]. The results showed a change in the behavior of the GF chains upon incorporating the emulsions. This change is reflected in the increased intensity of characteristic signals, such as those at 3200–3500 cm^−1^, which correspond to the matrix, and those at 1700–1500 cm^−1^, associated with amine groups. These changes could indicate interactions between the amino acids that make up the matrix and the active ingredients, as well as the appearance of new signals, suggesting interactions between the different components of the emulsions and the biopolymer.

### 3.8. Cytotoxicity and Wound-Healing Assay

**Figure 7:**
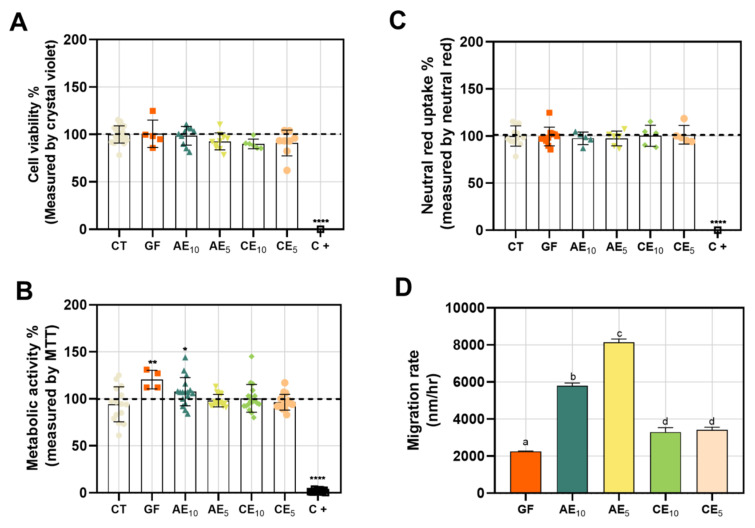
In vitro study in HaCaT cell line. (**A**) Cellular viability, (**B**) MTT, (**C**) neutral red uptake, and (**D**) migration rate determination in wound-healing assay. Results are presented as mean ± SEM for statistical analysis. Statistical analysis was performed using one-way ANOVA and the Kruskal–Wallis test (* *p* < 0.01; ** *p* < 0.005; **** *p* < 0.0001). For graph (**D**), identical letters in the reported data within each column indicate no significant differences.

Keratinocytes are cells found in the outermost layers of the skin, making them the primary recipients of damage in the event of an injury. Therefore, they play essential roles in the various stages of the healing process (hemostasis, inflammation, proliferation, and remodeling) [45]. They have a cross-activation system of the immune system through membrane receptors and pathogen recognition, and they send pro-inflammatory signals that activate cell proliferation and maturation, which are crucial for wound healing [46]. This is why, when designing a treatment for skin injuries, it is important to evaluate the cytotoxicity of the potential treatment on this type of cells. Upon evaluating different films in HaCaT cell cultures, we observed that they did not cause a loss of cell viability as determined by CV (Figure 7A). Additionally, there were no changes in cellular metabolism measured by MTT, except for the base film (GF), which caused an increase in mitochondrial activity, possibly attributable to the protein-based matrix of the film (Figure 7B). This could be over-nourishing the cells, leading to heightened metabolic activity. The integrity of the plasma membrane showed no damage that would prevent the incorporation of neutral red into its structure (Figure 7C). As a positive control for cell toxicity, EDTA 15 mM was used, known from the literature as an anion chelator that induces cell death. In line with this, we observed that this control affected cell viability, metabolic activity, and membrane integrity in the three assays conducted.

To evaluate if the films promote cell migration, facilitating wound closure in vitro, a gap was created in HaCaT cell layers, and then the cell migration rate was calculated using the ImageJ software based on images taken at 0 hpi and 24 hpi. The active film AE_5_ demonstrated the fastest wound closure, followed by the active film AE_10_, the films without active ingredients (CE_10_ and CE_5_), and finally, the base film (GF) (Figure 7D). These results outperformed other biopolymer-based wound-healing developments, such as the chitosan films by [47,48] polymeric films, which also showed lower wound closure efficiency compared to our active films. This indicates that the active films effectively promote wound closure, highlighting their potential use in human wound healing.

### 3.9. Toxicology Study in Zebrafish Model

**Figure 8:**
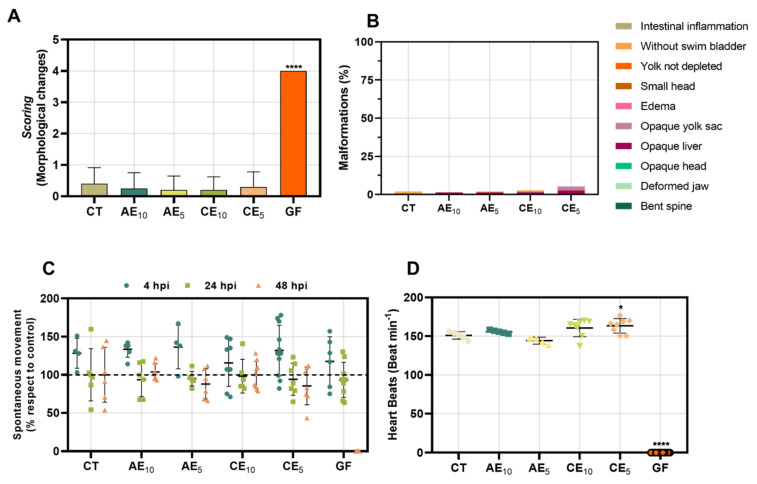
Toxicology study. (**A**) General toxicity; results are shown by scoring of morphological changes and (**B**) percentage of malformations. (**C**) Neurotoxicity and (**D**) cardiotoxicity. Results are presented as mean ± SEM for statistical analysis. Statistical analysis was performed using one-way ANOVA and the Kruskal–Wallis test (* *p* > 0.01; **** *p* < 0.0001).

The in vivo toxicity analysis of the different films was evaluated at 4, 24, and 48 h post-incubation (hpi). The films showed no mortality in any of the tested larvae except for the base GF, which was lethal at 48 hpi with 100% mortality. Although this result was unexpected, the films incorporating the different emulsions improved the performance of the base film in the toxicological studies. The morphological changes were studied at 48 hpi (Figure 8A,B). The films containing the emulsions did not cause marked morphological changes under the different conditions, unlike the GF, which presented the highest score, without being able to differentiate which anomalies were present specifically; nevertheless, these anomalies led to the absence of heartbeats. For the films with emulsions, the main detected effects were the presence of an opaque liver and the absence of the swim bladder. However, these effects were observed in a low percentage of individuals, showing a minimal impact on the population.

The study of spontaneous swimming in zebrafish larvae is intrinsically linked to neuronal activity, making this model an important tool for studying neurotoxicity. This is because, at 48 h post-fertilization (hpf), the larvae have developed neurons, a spinal cord, and neurotransmitter projections that are in their optimal functioning state when testing a treatment [49,50]. When evaluating the neurotoxic effects of our films, it was determined that GF induces a deterioration of locomotor activity, which progresses as the incubation time increases, with 100% loss of movement at 48 hpi (Figure 8C). However, this effect cannot be attributed to neurotoxicity, as the larvae were dead by the end of the study. It may be due to a combination of symptoms that led to the total deterioration of the larvae. On the other hand, when AE and CE films were compared, there was no marked difference in behavior, a conclusion reinforced by statistical analysis; analyzing the differences between conditions, they did not show significant differences.

Since most medications are processed in the liver, studying treatments’ effects on this organ is crucial. Therefore, this study examined the preservation of liver anatomy and its functions. It revealed that some larvae exhibited opaque areas in the liver at 48 hpi, indicating a potential future development of total tissue necrosis when exposed to AE and CE films.

Therefore, evaluating the effect on heartbeats of the films (Figure 8D), although it is a topical application, its potential disposal and environmental impact imply that studying this animal model is of vital interest. The treatment of the films was compared with the control, showing that the AE films did not exert a cardiotoxic effect on the larvae at 48 hpi under any conditions. However, arrhythmia was observed in larvae treated with CE_5_ films, with increased heartbeats. Finally, as seen previously, the GF showed a total absence of heartbeats due to lethality, resulting in 100% larval death.

Toxicological studies in this model showed that the GF exhibited acute toxicity, associated with the progressive deterioration of the larvae and their death at 48 hpi. However, these effects were not observed with the incorporation of emulsions in the AE and CE films, making this system a promising candidate for potential human biocompatibility and environmental friendliness.

## 4. Conclusions

An innovative composite system was developed based on gelatin as a matrix and O/W emulsions as carriers containing silver nanoparticles, sulfadiazine, lidocaine, and vitamin A as healing agents to treat skin lesions. This novel film offers a sustainable protective delivery system of active compounds and stable medium, supporting our main goal of diminishing the frequency of dressing exchange, avoiding the risk of infection, and mainly reducing periodic clinic visits. The water vapor permeability results indicated that the formulated films are suitable for wound dressings. Mechanical properties were affected by the addition of the emulsion, but no detrimental changes were found. In vitro studies indicated that the developed films do not exhibit cytotoxicity in a skin cell line and, importantly, promote a higher rate of cell migration for wound closure. In vivo assays demonstrated that the films are not toxic to zebrafish larvae, positioning this development as a potential candidate for application in skin wounds. Based on the results, our best formulation was AE_5_, which exhibited the best physicochemical properties. Notably, since zebrafish are environmental bioindicators, the fact that the films resulted in non-toxicity is essential for their use and eventual disposal.

## Figures and Tables

**Table 1:** Description of gelatin-based films.

Sample Names	Description
GF	Films based on gelatin without emulsion
CE_5_	Films based on gelatin with CE without active compounds (5:1 mass ratio, film dispersion/emulsion)
CE_10_	Films based on gelatin with CE without active compounds (10:1 mass ratio, film dispersion/emulsion)
AE_5_	Films based on gelatin with AE with active compounds (5:1 mass ratio, film dispersion/emulsion)
AE_10_	Films based on gelatin with AE with active compounds (10:1 mass ratio, film dispersion/emulsion)

**Table 2:** Mechanical parameters of films. The same letters in the data reported in columns mean non-significant differences. Statistical analysis was performed using one-way ANOVA followed by Tukey’s post-test (*p* ≤ 0.05).

Sample	TS (MPa)	YM (MPa)	ε (%)	Thickness (mm)
GF	2.2 ± 0.4 ^a^	0.04 ± 0.01 ^a^	46 ± 13 ^a^	0.042 ± 0.003 ^a^
AE_10_	3.6 ± 0.6 ^ab^	1.7 ± 0.4 ^b^	257 ± 20 ^b^	0.053 ± 0.003 ^b^
CE_10_	3.9 ± 0.6 ^b^	1.7 ± 0.5 ^b^	338 ± 21 ^c^	0.057 ± 0.002 ^b^
AE_5_	6.2 ± 0.5 ^c^	7.6 ± 0.8 ^c^	302 ± 32 ^c^	0.075 ± 0.002 ^c^
CE_5_	2.6 ± 0.8 ^a^	3.9 ± 0.7 ^d^	306 ± 20 ^c^	0.075 ± 0.005 ^c^

**Table 3:** Water vapor permeability of films. A statistical analysis was conducted using one-way ANOVA followed by Tukey’s post hoc test (*p* ≤ 0.05). Identical letters in the reported data within each column indicate no significant differences.

Sample	P_w_ 10^−10^(g s^−1^ m^−1^ Pa^−1^)	Thickness (mm)
GF	1.43 ± 0.02 ^a^	0.032 ± 0.03 ^a^
AE_10_	2.50 ± 0.01 ^b^	0.053 ± 0.003 ^b^
CE_10_	2.34 ±0.01 ^b^	0.057 ± 0.002 ^b^
AE_5_	2.70 ± 0.01 ^b^	0.075 ± 0.002 ^c^
CE_5_	2.60 ± 0.02 ^b^	0.075 ± 0.005 ^c^

**Table 4:** Initial and maximum degradation temperatures corresponding to the films. Temperature error: ±1 °C.

Formulation	T_i_ (°C)	T_max1_ (°C)	T_max2_ (°C)	T_max3_ (°C)
GF	145	280	305	-
AE_10_	140	325	385	435
CE_10_	160	270	380	440
AE_5_	155	310	390	431
CE_5_	180	310	345	400

**Table 5:** Glass transition temperature (Tg, °C) and endothermic transition (ΔH, J/g).

Formulation	Tg (°C)	ΔH (J/g)
GF	57	4.236
AE_10_	62	5.043
CE_10_	62	4.835
AE_5_	67	6.758
CE_5_	65	6.653

## Data Availability

The data from the publication can be obtained by requesting it from one of the authors.
